# The oxygenation module: the missing link in using sleep apnea devices to treat COVID-19 pneumonia at home

**DOI:** 10.1186/s12938-022-00982-z

**Published:** 2022-02-04

**Authors:** Dmitrijs Bliznuks, Svjatoslavs Kistkins, Jevgēnijs Teličko, Vadims Geža, Ģirts Zāģeris, Artis Svaža, Emil Syundyukov, Mārtiņš Purenkovs, Dana Zeme, Solveiga Jēkabsone, Dace Žentiņa, Valdis Pīrāgs, Immanuels Taivans

**Affiliations:** 1 Institute of Smart Computer Technologies, Riga Technical University, 2-335 Daugavgrivas street, Riga, 1658 Latvia; 2 Department of Internal Medicine, Paul Stradins Clinical University Hospital, 13 Pilsonu street, Riga, 1002 Latvia; 3 University of Latvia Institute of Numerical Modelling, 3 Jelgavas street, Riga, 1004 Latvia; 4 Paul Stradins Clinical University Hospital, Research Institute, 13 Pilsonu street, Riga, 1002 Latvia; 5 Faculty of Computing, University of Latvia, 19 Raina bulvaris, Riga, 1586 Latvia

**Keywords:** Sleep apnea, COVID-19, Critical Care, Non-invasive ventilation, Homecare

## Abstract

**Background:**

The study aims at solving the problem with the limitations of the homecare CPAP equipment such as sleep apnea devices in the treatment of COVID-19 pneumonia. By adding an advanced, rapid-to-produce oxygenation module to existing CPAP devices we allow distributing healthcare at all levels, reducing the load on intensive care units, promoting treatment in the early stages at homecare. A significant part of the COVID-19 pneumonia patients requires not only an oxygen supply but also additional air pressure. Existing home care devices are able to create precise positive airway pressure, but cannot precisely measure supplied oxygen concentration. Either uses uncertified and potentially unsafe mechanisms.

**Results:**

The developed system allows using certified and widely available CPAP (constant positive airway pressure) devices to perform the critical function of delivering pressure and oxygen to airways. CPAP device is connected to the designed add-on module that can provide predefined oxygen concentration in a precise and stable manner. Clinical test results include data from 12 COVID-19 positive patients. The device has been compared against certified NIV (non-invasive) equipment under 6–20 hPa pressure and 30–70% FiO_2_. Tests have proved that the developed system can achieve the same SaO_2_ (*p* = 0.93) and PaO_2_ (*p* = 0.80) levels as NIV with clinically insignificant differences. Test results show that the designed system can substitute NIV equipment for a significant part of COVID-19 patients while leaving existing NIV devices for unstable and critical patients. The system has been designed to be mass-produced while having medically certified critical components.

**Conclusion:**

The clinical testing of the new device for oxygen supplementation of patients treated using simple CPAP devices looks promising and could be used for the treatment of COVID-19 pneumonia.

## Background

In the majority of cases, COVID-19 runs with inflammation in the upper airways and manifests with symptoms like dry cough, subfebrile or febrile temperature, and resolves in several days. Some patients [1] develop pneumonia with symptoms of hypoxia and decreased level of SpO_2_. Therefore, the management of COVID-19 caused type 1 respiratory failure patients includes the administration of additional oxygen and prevention of distal airway and alveolar collapse [2, 3]. These options can be maintained by using non-invasive ventilation (NIV) applying positive end-expiratory pressure (PEEP). The existing intensive care NIV devices and high flow nasal cannula (HFNC) currently are used in treatment type 1 respiratory failure in patients with COVID-19 pneumonia [4–6]. However, shortages in the number of available NIV or HFNC, as well as high costs of the devices, provide several significant limitations, especially during the outbreak when time and expenses are crucial [7]. For this purpose, a cheap and rapidly produced solution must meet the criteria of clinical efficacy similar to the efficacy of NIV or HFNC.

The simplest way to promote PEEP is to use devices that are designed for the treatment of sleep apnea syndrome—continuous positive airway pressure (CPAP) devices that keep slightly positive pressure in airways, so preventing airways collapse at the end of expiration. These instruments also allow the administration of additional oxygen by introducing it into the inspiratory port or directly into the face mask [8].

However, this oxygen administration does not allow precise measurement of oxygen concentration in inhaled air because of fluctuations of oxygen level due to continuous air movements in the inspiratory port. At the same time a proper oxygen concentration is significant for the calculation of alveolar-arterial gradient for oxygen, which is an indicator of permeability of the alveolo-capillary membrane, or in other words—an indicator of the level of lung respiratory function loss. Continuous measurement of this index allows monitoring the clinical condition, providing rapid response in deterioration of the disease [9].

Therefore, we have created the add-on device for CPAP instruments allowing us to deliver the precise oxygen concentration to the patient. The study aimed to modify CPAP devices and clinically investigate the efficacy of the proposed solution by comparing it with current existing NIV devices.

During the COVID-19 pandemic there was a rapidly increased demand on ICU facilities, especially mechanical lung ventilators (MVL). The shortage of MLV raised the question of early CPAP treatment in COVID-19 pneumonia patients. In CPAP, additional pressure is provided to the alveoli, which prevents them from collapsing at the end of the exhalation [1, 2]. This results in improved gas exchange, reduced breathlessness, and respiratory rate. Also, CPAP might protect the patient from the further progression of lung damage and lower the tracheal intubation rate. In addition, it is possible to reduce oxygen concentrations (FiO_2_) in the inhaled air by using positive pressure, thereby minimizing the toxic effects of oxygen and utilizing oxygen capacity more cost-effectively [3, 4]. Until the beginning of the COVID-19 pandemic, CPAP equipment was used to treat obstructive sleep apnoea at home. The use of CPAP devices is now also introduced for the treatment of acute patients in hospitals [5]. The amount of oxygen to be delivered by the CPAP can only be controlled by the oxygen supply rate following the CPAP device. This may help the medics decide on the future respiratory failure therapy tactics for a patient with hypoxaemia, thereby making CPAP a promising option in a hospital setting.

**Fig. 1:**
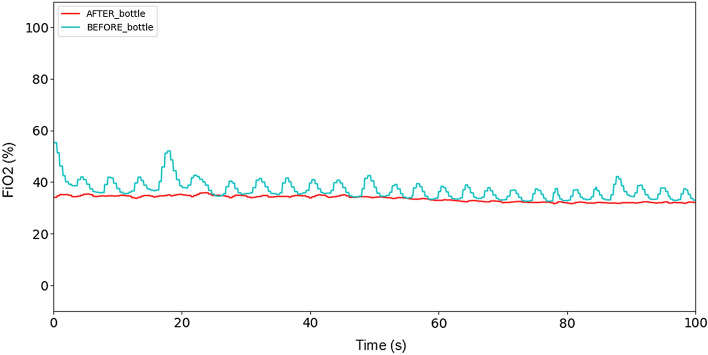
FiO_2_ measurements in two oxygen sensor locations: before (blue line) and after (red) the buffer tank under 6 hPa pressure

The idea of the study is based on the results of early CPAP therapy described in a study published by BMJ Open Respir Res [5]. In Fig. 1, the probability of survival was rapidly reduced after day 4 of treatment for patients included in the study.

It's worth noting diabetes and arterial hypertension as risk factors for disease progression. The study compared mortality in patients who received CPAP therapy at the early phase of the disease (before the seventh day) and at late phase (after the seventh day). Mortality appeared to have decreased significantly in patients with diabetes (19% vs 43%) and arterial hypertension (8 vs 26%). The rapid progression of the disease correlated with the radiological scene of pneumonia when the ground-glass phase (days 1–6) transforms into the reticulation phase (7–14). Early treatment with CPAP was also described in other studies [6, 7] as well as in editorial letters[8]. The letter *to ERJ* Journal [9] noted the stratification of patients was made based on radiological manifestation, also the majority of patients received relatively early (days 3–10) CPAP therapy. Although studies prioritize non-invasive lung venting (NIV) treatment, it is still challenging to identify technical parameters and inclusion criteria that would influence the outcome [10–12].

For example, researchers correlate high levels of FiO_2_ (0.4–0.8) or high oxygen use (> 10 L/min) before using NIV with higher mortality (OR 1.59) [13, 14]. In turn, for those who used non-invasive ventilation with lower FiO_2_ support, mortality decreased [15]. *RECOVERY-RS*, one of the most important studies, compared conventional oxygen therapy and HFNC with CPAP [16].Currently, the new data from *RECOVERY-RS* has been published in the preprinted form and is awaiting comments from reviewers. However, taking into account all the above data, the results show that the CPAP group has significantly lower mortality and a transition to MLV compared to conventional or HFNC therapy.

## Results

### Bench testing

As shown in the methods section, after performing numerical simulations of oxygen mixing performance, the final design with the buffer tank has been used for building a prototype. In Fig. 1 oxygen concentration with and without buffer tank can be seen. The resulting oxygen concentration fluctuations with a buffer tank were < 1%.

The plot has been obtained by using a prototype device set at 6 hPa positive pressure and a healthy person. While the mask is properly attached and there are no breathing abruptions, the system could keep oxygen concentration accuracy within 1%, even if the character of the patient's breathing changes. Without a buffer tank oxygen concentration changes according to breathing frequency. Right after switching the system or after reapplying the mask, it might take up to a minute, while concentration reaches the desired level and stabilizes. Such effects do not affect therapy and are acceptable as seen in the clinical results section below.

### Clinical testing in patients with COVID-19 pneumonia

Totally 16 patients with COVID-19 pneumonia have been enrolled on a clinical trial and 12 of them have been included. The characteristics of patients included in a clinical trial are shown in the table below. Two patients have been excluded due to intolerance to a face mask, while the third patient has been expelled due to clinical deterioration receiving primarily only NIV. The fourth patient has been excluded due to arterial catheter thrombosis.

To compare the effectiveness of the new oxygen supply system (CPAP+) with a standard non-invasive ventilator (NIV) we used a repeated-measures analysis of variance (ANOVA).

**Fig. 2:**
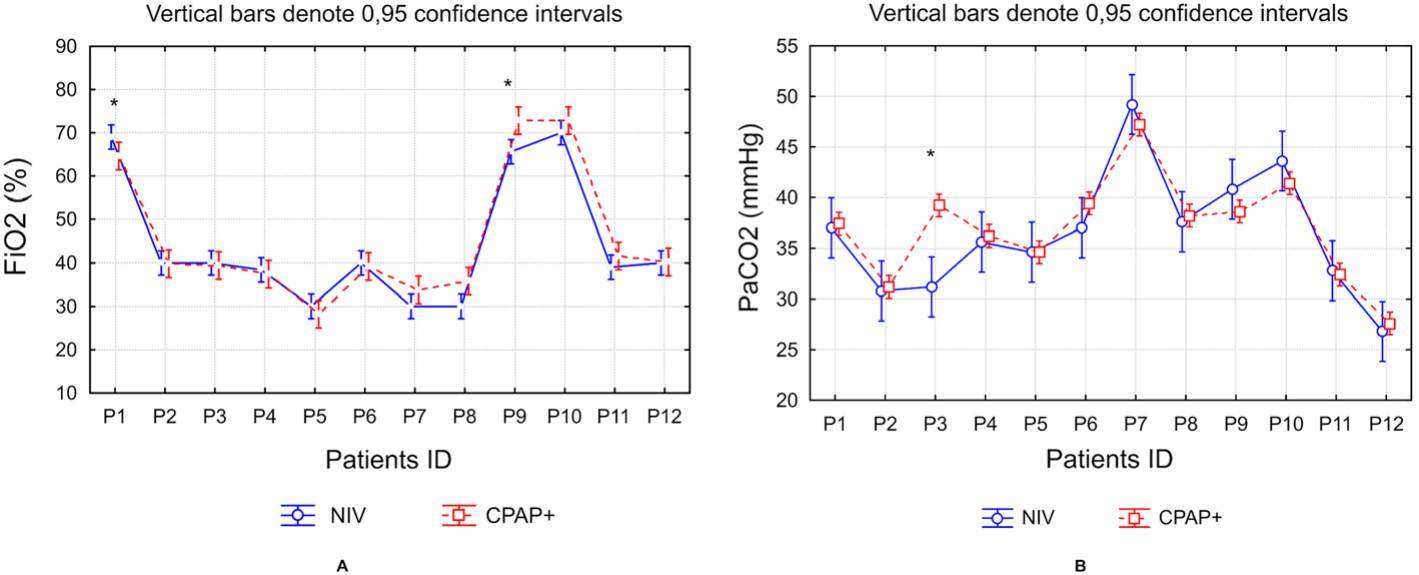
**A** FiO_2_ levels necessary to keep oxygen saturation at normal levels in individual patients using two oxygen delivery systems. **B** PaCO_2_ levels in individual patients treated with two compared devices

Figure 2A allows comparing necessary concentrations of oxygen in the inhalation line (FiO_2_) which were set on two devices to keep the stable oxygen saturation (SaO_2_). The Fig. 2 shows that FiO_2_ levels did not differ significantly in the majority of patients. Only in two cases when changing from one instrument to another different oxygen concentration had to be set. Figure 2B reflects the levels of carbon dioxide measured in arterial blood (PaCO_2_) Graph shows that two patients, P7 and P10 had CO_2_ concentrations above 40 mmHg, indicating carbon dioxide retention. At the same time breathing with aid of any of both devices did not change high CO_2_ values. A significant difference between CO_2_ values (Fig. 2B) in patient 3 probably occurred due to hypocapnia induced by hyperventilation and did not depend on the device used.

**Fig. 3:**
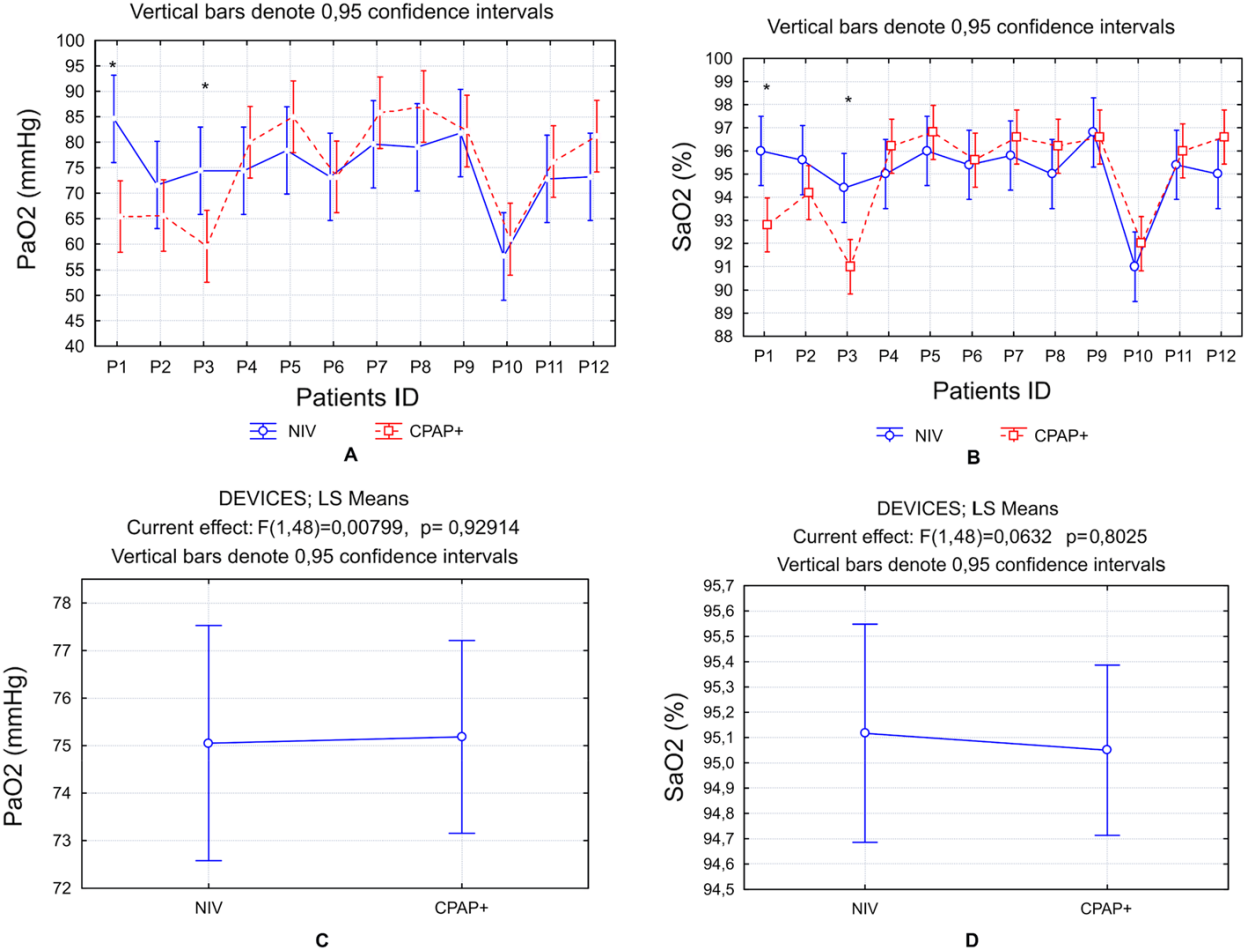
**A** PaO_2_ levels in individual patients under two testing conditions. **B** SaO_2_ levels in individual patients under two testing conditions. **C** Mean PaO_2_ values in all patients cohort breathing by the aid of two compared devices. **D** Mean SaO_2_ values in all patients cohort breathing by the aid of two compared devices

Most precisely the effectiveness of oxygen supplementation may be checked by analyzing arterial blood gases. The most sensitive index is partial oxygen pressure. Figure 3A, B demonstrate partial pressure of oxygen in arterial blood (PaO_2_) levels in individual patients being connected to both tested devices. Significant differences in PaO_2_ and SaO_2_ levels have been presented only in two patients. In these cases, a lower level of PaO_2_ has been observed in the trial with the CPAP+ device. However, as can be seen from the graph, in other patients the situation can be the opposite—higher levels of PaO_2_ with CPAP+ device. Taken together, the comparison between two devices in all patients cohorts show no significant differences between PaO_2_ and SaO_2_ values (Fig. 3C, D).

**Fig. 4:**
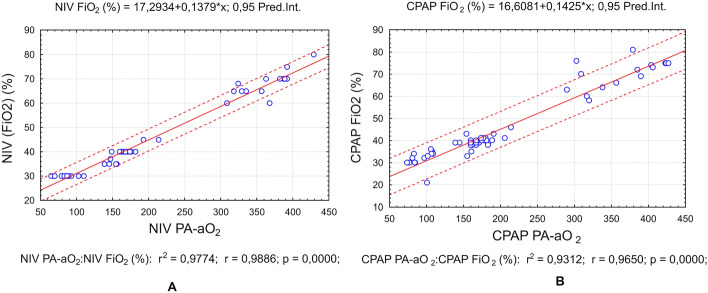
Regression line with predictive intervals demonstrating the dependence of FiO_2_ from AaDO_2_. Data obtained from NIV (**A**) and CPAP (**B**) devices

The necessary FiO_2_ for each patient depended on the severity of their disease. A precise indicator of the effectiveness of gas exchange is the alveolar-arterial gradient of oxygen (AaDO_2_). Figure 4 shows the regression analysis between FiO_2_ and alveolar-arterial gradient. Graphs representing data obtained with both devices (NIV—Fig. 4A; CPAP +—Fig. 4B) show high predictiveness levels.

Summary of significant differences between most important indices measured in two trials is presented in Table 1, showing *p* values. The full table of all measured and calculated indices is available in Annex.

**Table 1:** *p*-values characterizing the differences between the mean values of indices measured using NIV and CPAP + instruments in individual patients

Pac. ID	FiO_2_	SaO_2_	PaO_2_	PA-aO_2_	PaCO_2_
P1	**0.050**	**0.001**	**0.001**	0.136	0.801
P2	0.927	0.134	0.251	0.765	0.801
P3	0.785	**0.001**	**0.006**	0.787	**0.0001**
P4	0.649	0.198	0.284	0.722	0.704
P5	0.414	0.388	0.208	0.570	1.000
P6	0.716	0.829	1.000	0.776	0.135
P7	0.088	0.388	0.236	0.119	0.211
P8	**0.011**	0.198	0.128	**0.022**	0.705
P9	**0.002**	0.829	0.939	0.755	0.170
P10	0.206	0.282	0.514	0.190	0.170
P11	0.240	0.517	0.514	0.272	0.801
P12	0.927	0.088	0.128	0.842	0.614

Values indicating significant differences are marked with bold

## Discussion

The results of this study showed no statistically significant clinical differences between NIV and modified CPAP+, proposing a further discussion regarding the cost-effectiveness of the solution during the state of emergency.

It would be appropriate to compare currently existing three available types of devices in CPAP mode: the authors proposed home-care APAP/CPAP modification in comparison with two types of NIV device: hospital equipment and both hospital/home care devices.

In the table below (Table 2), CPAP+ modification is 4 to 15 times cheaper than alternative solutions. As the clinical study shows, device efficiencies are statistically similar to ICU-NIV. It is worth mentioning that the CPAP  modification has been clinically tested with only FiO_2_ < 70% while ICU-NIV equipment reaches 100% oxygen concentration. However, preclinical results showed FiO_2_ > 95%. It must be taken into account that we have aimed to develop a cost-effective device that can reduce the load on existing ICU-NIV devices.

**Table 2:** Comparison of three available types of devices in CPAP mode

	Our CPAP + device	NIV in hospitals (ICU−NIV)	NIV in hospitals + home care
Full set price in EUR	800	10,000–13,000	3000–7000
Oxygen supply from the central gas system without a flow meter	+	+	−
Oxygen supply from the central gas system with a flow meter, up to 15 l/min	+	+	+
Oxygen supply from low-pressure oxygen concentrators	+	−	+
Maximal achievable FiO_2_	70%—95%	100%	Depending on PEEP
Oxygen concentration is displayed on a screen	+	+	−
Ability to control and regulate oxygen concentration independently from the applied pressure	+	+	−
Necessary technical and electrical safety checks	Once a year	Once a year	Not required

The proposed benefits of the modification of the home CPAPs may be defined as low costs per one unit (800 EUR per home CPAP and add-on comparing to 10,000 EUR per ICU-NIV); rapid production and distribution (2–3 weeks vs 3–5 months); redirection of home-CPAP devices for the use in chronic conditions, such as sleep apnea after the outbreak. The potential cost-effectiveness may be significantly higher if the sleep apnea device could be rearranged for the patients that are primarily diagnosed with OSA. At the same time, strategic reserves of CPAP devices may be stored in hospitals to be ready for the new wave.

Industrial enterprises and scientists also tried to succeed in development of low-cost ventilators [11–15]. Despite the fact that Garmendia et al. [16] designed and clinically tested even much cheaper CPAP machines proposing 70–80 EUR instead of 600 EUR, the next study shows that early CPAP intervention could result in a potentially viable treatment option for patients only during the first days of hospitalisation [17]. Garmendia's CPAP device could be used in the early stage of the disease while moderate requires significant additional oxygen therapy [18]. Pandor et al. previously compared the cost-effectiveness of the prehospital-CPAP without a proper oxygen delivery arguing that the results of the study were uncertain [19]. The proposed additional solution may enhance the efficacy of the Garmendia CPAP devices, providing even cheaper analogues of NIV devices for more severe patients requiring higher oxygen concentration.

During the first outbreak Engineers of UK Formula 1 have developed a CPAP machine with the distribution of oxygen and positive airway pressure to patients [20, 21]. The F1 CPAP device mixes gases in a proper concentration based on the Venturi effect thus resulting in a proper gas formation with much predictable FiO_2_ concentration. However, this device also requires an additional outsourced oxygen sensor and a pressure valve promoting 15–20 mmH_2_O pressure. A cost-effective solution met several disadvantages such as high oxygen expenditures due to the construction of the equipment with a further load on the oxygen supply chain in the hospital. A high oxygen expenditure promotes overload of hospital medical gas system [22, 23]. As the experience showed, frequently CPAP equipment can be used at home, additionally relieving the medical infrastructure [24, 25]. Moreover, it facilitates saving of financial means, because the acquisition of a large number of intensive therapy lung ventilators is not needed. For this purpose the proposed CPAP modification can use an external oxygen delivery system such as oxygen concentrators, reducing the load on the oxygen supply chain in the hospital.

Despite the advantage of this method of oxygen delivery there were some observations and raising questions about the usage of the device and its operation:

As mentioned in the previous sections, the device has accuracy limitations during sudden flow changes, for example reapplying the mask. The device buffer tank volume has been calculated to smooth fluctuations during inhale and exhale phase when flow change stays below a certain limit. If the mask is removed, flow increases rapidly and since oxygen flow is limited, the buffer is emptied fast and oxygen concentration drops below a predefined level. The same effects could be observed if the mask is not applied properly and there are occasional false-air situations. Existing NIV devices premix oxygen before performing pressure/flow control therefore can keep constant concentration-independent to the mask application.

Other limitations of the developed system include the inability to transport patients since the device has no autonomous power source. The device is not delivering additional breathing measurements like ICU ventilator: tidal volume, minute ventilation, peak pressure, flow, volume, and pressure waves. The current design requires significant time to dismount air pathways for sterilization. Nevertheless, sterilization by using special gases is possible.

Clinical testing of the new CPAP supplemental oxygen delivery system has shown that this device allows enriching the air with oxygen up to 70% during CPAP therapy (higher concentrations have only been tested pre-clinically). Comparison with a standard non-invasive ventilator (NIV) working in CPAP mode has shown that most important indices of gas exchange, like PaO_2_ and PaCO_2_, did not change significantly when switching the patients from breathing with aid of NIV instrument to CPAP+.

Two cases (P1 and P3) showing significant differences in PaO_2_ between two devices evidently have been connected with changes in the patient’s condition and did not depend on the instrument. Testing of the patient P1 was started with NIV instrument. FiO_2_ level was set on 80% oxygen that corresponded to previous necessary level for this patient. After 15 min breathing arterial PO_2_ level raised to 102 mmHg and saturation to 98% that was obviously too high. Therefore, O_2_ concentration was corrected to the level of 70% and thereafter to 60%. By the end of the test period PaO_2_ was 70 mmHg and SaO_2_—94%. Testing with CPAP+ instrument was started with FiO_2_ level of 60% and gradually raised to 65% to keep the O_2_ saturation at optimal level. These changes explain statistically significant differences between mean PaO_2_ values in these two trials. The situation was similar with patient P3.

The patient’s need for additional oxygen changes over time. Most likely cause is the ventilation/perfusion mismatch that occur with changing the position of the chest [26].

Significantly lower levels of PaCO_2_ in patient 3 when breathing through a NIV device, are likely to be associated with anxiety-induced hyperventilation at the start of the experiment. This was also reflected in the respiratory rate which was higher at the beginning of the test. When treated with CPAP+ device, the patient was calm and his PCO_2_ level was in the normal range.

## Conclusions

The results of this study showed no statistically significant clinical differences between NIV and modified CPAP+ in case of FiO_2_ < 70%. Elaborated oxygen supplementation module expands the range of COVID-19 patients to be treated with CPAP devices. This solution is significantly cheaper than NIV devices and can be used in both hospital and home settings. Further clinical studies are necessary to provide long-term safety and efficacy data before the system is applied to routine clinical practice.

## Methods

### Ventilator description

**Fig. 5:**
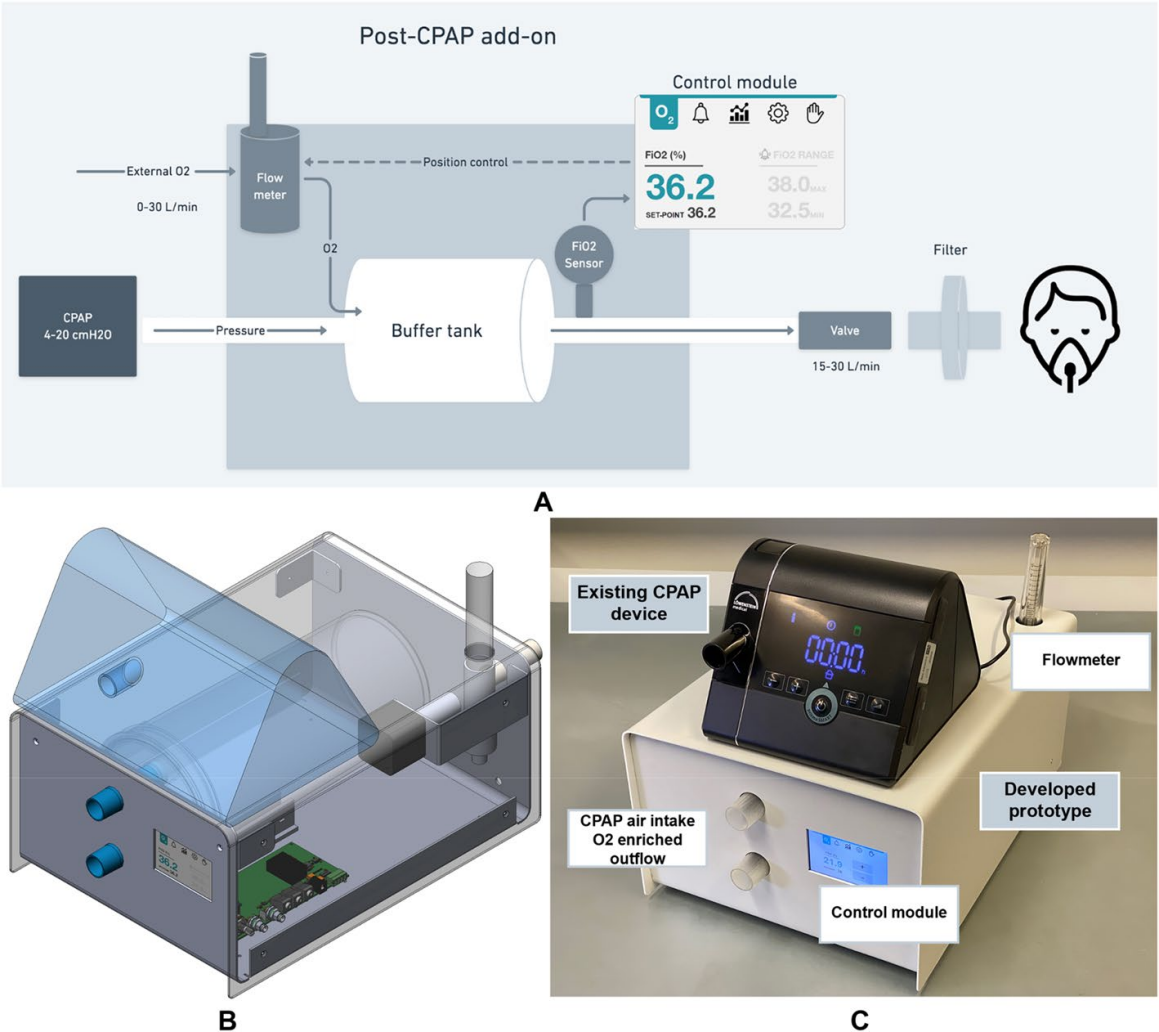
**A** The scheme of the modification of a certified CPAP device. **B** Device 3D model **C** The final metal enclosure with CPAP device on top

The proposed device has been designed as an add-on to the CPAP device that is certified to provide an automatically controllable air pressure supply to the patient. The overall scheme of the modified system is shown below in Fig. 5A.

The device allowed controlling oxygen supply to the patient’s inhaled air in the FiO_2_ range of 21% to 95%. Supplied air pressure and moisture have been controlled by a certified CPAP device. During the development stage, two versions of the CPAP add-on have been designed: draft prototype to prove the concept (Fig. 5B) and final prototype ready to be mass-produced (Fig. 5C).

The microcontroller-based system monitors oxygen concentration at the output of the tank and adjusts the oxygen supply to keep the FiO_2_ at the set level. Clinicians can see the current FiO_2_ level on the LCD touchscreen and can change it according to therapy. The device has alarm and logging capabilities to monitor patients' condition and store data for further analysis. The device applies to Type B (Body) class—“No direct physical contact with the patient”. And to the product class IIA as for “Directive 93/42/EEC”. The device served as an add-on to the existing certified CPAP equipment (eg. Löwenstein medical Prisma Smart). The add-on has been electrically operated, with no direct contact with patients or operators (health care personnel). Oxygen flow control has been provided by using a medical-grade flow meter as an intermediate device. Stepper motor was used to rotate the flowmeter valve that in a result changes oxygen flow. Stepper motor is controlled by software PID controller that uses FiO_2_ sensors data for feedback. To make sure that sensors readings are correct, two FiO_2_ sensors were used and if the difference in their reading was larger than threshold, system gave an alarm.

To obtain a controllable oxygen supply to the patient, it is essential to do precise measurements of oxygen concentration in the flow. However, oxygen concentration may vary significantly across the cross-section of the flow channel (pipe) if gases are not premixed. For this reason, numerical simulations were carried out for different mixing setups to establish a flow where oxygen concentration is precisely measurable. The simplest way to supply oxygen is to make a straight oxygen pipe connection to the main CPAP flow channel. Numerical simulation results of oxygen concentration reveal high inhomogeneity. X-type mixer appropriately performs in the terms of oxygen homogeneity, but it greatly increases hydraulic resistance (+ 15%). Similar questions have also been raised in literature adding a viral/bacterial filter proximally to patient [10]. To deal with this problem, a custom mixer has been designed that allowed mixing oxygen with homogeneity > 99.5% and low hydraulic resistance (+ 5%). An additional challenge for the add-on device creation was reaching stable concentration levels of oxygen at any breath phase. That was achieved by the incorporation of a buffer tank into the inhalation line. Multiple simulations have been performed to find an optimal volume and shape of the tank.

### Ventilator testing in patients with COVID-19 pneumonia

The clinical testing of the new device has been realized by comparing the efficiency and safety of the new CPAP supplemental oxygen delivery system with the efficiency of a non-invasive ventilator (NIV) in the CPAP/PEEP mode. Two Intensive care ventilators have been used—*Philips Respironics V60 and Maquet Servo—AIR in CPAP mode* for one hour and then changed therapy to *APAP Lowenstein medical Prisma Smart* connected to newly made extra oxygen delivery device.

Hypoxaemic patients, who received just oxygen therapy or NIV therapy in CPAP mode have been included in the study.

The study was evaluated and received permission from the local Ethics committee of the PSCUH. Patients have been informed that a catheter will be inserted into their artery to get blood samples. The patient’s characteristics are given in Table 3.

**Table 3:** Patients’ data

Patient ID	Gender	Age, years	SpO_2_	AaDO_2_	PaCO_2_	PEEP
P1	M	80	95	372	37	5
P2	M	77	94	175	31	5
P3	M	63	94	171	31	9
P4	M	44	97	155	36	5
P5	M	42	95	92	35	6
P6	M	44	95	165	37	5
P7	F	33	98	73	48	6
P8	M	53	96	87	38	5
P9	M	53	95	333	41	7
P10	M	62	92	388	44	12
P11	F	79	96	163	33	6
P12	M	78	95	178	27	5

The majority of patients were males (83%). The table shows that patients were selected in stable condition, their transcutaneous oxygen saturation was kept at least above 92% by adjusting both FiO_2_ and PEEP at necessary levels.

Inclusion criteria were age > 18, hypoxaemia < 92% and < 88% in patients with risk of hypercapnic respiratory failure. Exclusion criteria were contraindications for non-invasive ventilation (e.g. unstable conditions of upper airways, aspiration risks) and clinical deterioration of patient condition that was decided by study performing physician.

Before the experiment, the arterial catheter has been inserted into a patient's radial artery and the first arterial blood probe has been taken after hypoxaemia was corrected. The examination lasted one hour, each 15 min the blood probe was repeated and checked for ABG parameters. Straight after or after 15-min break patients have been connected to the new CPAP supplemental oxygen delivery system and FiO_2_ and PEEP levels have been set similar to the previous instrument. FiO_2_ and PEEP levels, if necessary, were corrected regarding arterial blood gas analysis and SpO_2_ measurements.

SpO_2_ has been monitored continuously, blood gas analysis has been repeated every 15 min (pH, PaO_2_, PaCO_2_, HCO_3_–, SaO_2_), blood pressure, heart rate, respiratory rate, with the help of an additional sensor FiO_2_. With each subsequent device, the PEEP size has been selected the same as with the previous device or its modification.

Research data has been processed using ‘MS Excel’ and ‘SPSS’ software by repeated-measures analysis of variance (‘ANOVA’). The obtained data (pH, PaO_2_, PaCO_2_, HCO3−, SaO_2_, blood pressure, heart rate, respiratory rate, with the help of an additional sensor FiO_2_) have been compared between all groups.

## Data Availability

Not applicable.

## Abbreviations

AaDO_2_: Alveolar-arterial gradient of oxygen
ABG: Arterial blood gases
APAP: Automatic positive airway pressure
COVID-19: Coronavirus disease 2019
CPAP: Continuous positive airway pressure
FiO_2_: Fraction of inspired oxygen
HFNC: High flow nasal cannula
ICU: Intensive care unit
LCD: Liquid–crystal display
NIV: Non-invasive ventilation
OSA: Obstructive sleep apnea
PaO_2_: Partial pressure of oxygen in arterial blood
PaCO_2_: Partial pressure of carbon dioxide pressure in arterial blood
PEEP: Positive end-expiratory pressure
PID: Proportional-Integral-Derivative (controller)
PSCUH: Paul Stradins Clinical University Hospital
SaO_2_: Arterial oxygen saturation
SpO_2_: Oxygen saturation provided by pulseoxymetry
